# Fungal biological resources to support international development: challenges and opportunities

**DOI:** 10.1007/s11274-019-2709-7

**Published:** 2019-08-26

**Authors:** M. J. Ryan, K. McCluskey, G. Verkleij, V. Robert, D. Smith

**Affiliations:** 1 CABI, Bakeham Lane, Egham, TW20 9TY Surrey UK; 2 Bolt Threads, Emeryville, CA 94608 USA; 3 Westerdijk Fungal Biodiversity Institute, P.O. Box 85167, 3508 AD Utrecht, The Netherlands

**Keywords:** Fungi, Cryopreservation, Microbiome, Nagoya, Bioinformatics, Taxonomy

## Abstract

Exploitation of microbes, especially fungi, has the potential to help humankind meet the UN’s sustainable development goals, help feed the worlds growing population and improve bioeconomies of poorer nations. The majority of the world’s fungal genetic resources are held in collections in developed countries, primarily within the USA, Europe and Japan. Very little capacity exists in low to middle income countries, which are often rich in biodiversity but lack resources to be able to conserve and exploit their own microbial resources. In this paper we review the current challenges facing culture collections and the challenges of integrating new approaches, the worth of collaborative networks, and the importance of technology, taxonomy and data handling. We address the need to underpin research and development in developing countries through the need to build ‘in country’ infrastructure to address these challenges, whilst tackling the global challenges to meet the requirements of the research community through the impacts of legislation and the Nagoya protocol on access to biological resources.

## Introduction and current status

International Biological Resource Centres underpin research and development and the global bioeconomy, and they are well placed to underpin infrastructure to aid governments as they strive to deliver their commitments to the UN’s sustainable development goals (SDG’s). However, to achieve this they must not only consolidate their existing capacities but evolve their approaches to meet the ever-changing requirements of their users. Many have broad remits while others have specialist functions and focus on specific groups of organisms. Microbial collections of living fungi, viruses, yeasts, and bacteria are spread throughout the world. Of the 739 collections listed on the World Data Centre for Microorganisms (WDCM), the biggest public service collections are located in developed countries in Europe, North America and East Asia (Table 1). While collections located in member countries of the Organisation for Economic Co-operation and Development (OECD) are relatively well established, there is a lack of provision in low to middle income countries (LMIC).

**Table 1:** Culture collections, in developed countries with significant fungal holdings

Collection (acronym)	Country/region	Coverage	Total Microbial holdings* (of which fungi and yeast)
American Type Culture Collection (ATCC)	USA—North America	Fungi, Bacteria, Yeast,	64,000 (46,000)
Belgian Coordinated Collections of Microorganisms/IHEM Fungi collection (IHEM)	Belgium—Europe	Fungi, Bacteria, Yeast,	14,722 (14,722)
BIOTECH (BCC)	Thailand—South East Asia	Fungi, Bacteria, Yeast,	80,000 (50,747)
CABI (IMI)	UK—Global	Fungi, Bacteria, Yeast,	30,000 (28,000)
Westerdijk Fungal Biodiversity Institute (CBS)	Netherlands—Europe	Fungi, Bacteria, Yeast,	100,000 (88,000)
Canadian Collection of Fungal Cultures, Agriculture and Agri-Food Canada	Canada—North America	Fungi, Yeast	17,030
Center for Fungal Genetic Resources	Korea—Asia	Fungi	24,531
China Center for Type Culture Collection	China—Asia	Fungi, Bacteria, Yeast, Plasmids, Cell lines, Viruses	21,985 (8000)
China General Microbiological Culture Collection Center	China—Asia	Bacteria, Fungi, Yeasts and cultures for patent purpose	53,906 (22,110)
EX Culture Collection of extremophilic fungi, University of Ljubljana	Slovenia—Europe	Bacteria, Fungi	12,350 (10,800)
Fungal Genetic Stock Center (FGSC)	USA—North America	Fungi & Yeast	29,000
International Collection of Microorganisms from Plants, Landcare Research	New Zealand—Oceania	Bacteria, Fungi	18,675 (9370)
Japanese Collection of Microorganisms (JCM)	Japan—East Asia	Fungi, Bacteria, Yeast,	24,772 (3371)
KCTC Korean Collection for Type Cultures	Korea—Asia	Bacteria, Fungi, Yeasts, Plasmids, Cell lines, Archaea, Microalgae, Patent	23,175 (6559)
IBT Culture Collection of Fungi, Technical University of Denmark	Denmark—Europe	Fungi	35,000
Microbial Culture Collection, Institution National Centre for Cell Science	India—Asia	Bacteria, Fungi	164,652 (15,338); Includes 150,000 bacterial strains isolated under Department of Biotechnology Microbial Prospecting project (Sharma and Shouche 2014)
Mycology Culture Collection, SA Pathology	Australia—Oceania	Fungi	10,000
NARO Genebank, Microorganism Section	Japan—East Asia	Bacteria, Fungi, Yeast, Protozoa, Viruses, Mycoplasmas, Nematodes	26,162 (19,807)
Nite Biological Resource Centre (NBRC)	Japan—East Asia	Fungi, Bacteria, Yeast,	27,906 (15,145)
Agricultural Research Service Culture Collection (NRRL)	USA—North America	Fungi, Bacteria, Yeast,	96,198 (73,702)
Phaff Collection (UCD-FST)	USA—North America	Yeast	7,270
UAMH Center for Global Microfungal Biodiversity	Canada–North America	Bacteria, Fungi, Yeast	13,080 (13,000)

^*^Yeast, bacteria and fungi, *Source* www.wdcm.org

Despite Africa being the world's second largest, second most-populous continent and biodiversity rich, with 54 recognised countries (OECD) there are just 17 WDCM registered collections, located in just 7 countries (Table 2). This leaves 47 countries without a registered collection. Of those countries which have collections, just two are fungal, while minor are general microbiology collections. These collections hold 28,650 strains, of which 89% are held by South Africa and Egypt.

**Table 2:** WDCM registered culture collections in Africa

Country	Collection	Acronym	# of organisms
Zimbabwe	Biological Sciences	BDUZ	160
Morocco	Moroccan Coordinated Collections of Microorganisms	CCMM	1582
Egypt	Culture Collection Ain Shams university	CCASU	20
Uganda	Uganda Trypanosomiasis Research Organization	EATRO	550
Egypt	Egypt Microbial Culture Collection	EMCC	1808
Senegal	Mircen Afrique Ouest	MAO	210
Zimbabwe	Grasslands Rhizobium Collection	MAR	537
Nigeria	Microbial Culture Collection in Enzymology and Protein Chemistry Unit, Department of Biochemistry (University of Nigeria)	MCCEPU	17
Egypt	Microbial factory	mf	11,700
Egypt	Microbiology Lab Culture Collection	MLCC	1
Egypt	MS Microbiology Ain Shams	MSASU	4
South Africa	South African Plant Pathogenic and Plant Protecting Bacteria	PPPPB	860
South Africa	National Collections of Fungi: Culture Collection	PPRI	10,000
South Africa	South African Rhizobium Culture Collection	SARCC	58
Egypt	Suez Canal University Fungarium	SCUF	1020
Nigeria	University of Calabar Collection of Microorganisms	UCCM	20
Nigeria	Department of Virology	VRLI	103
		Total	28,650

*Source* www.wdcm.org

Countries that do not have their own collections rely on those located in other regions. CABI, an international not-for-profit organisation owned by 49 member countries, offers deposit services globally. CABI acts as custodians of microbial genetic resources on behalf of its member countries, many of which are LMIC countries in Africa, Asia and the Caribbean. The collection holds 30,000 strains from diverse geographical holdings originating from over 140 countries. Of these, 2900 originate from Africa, the vast majority from countries that do not have the infrastructure to manage and operate their own culture collections. Other major collections such as the Westerdijk Fungal Biodiversity Institute (formerly the Centraalbureau voor Schimmelcultures - CBS) and the Fungal Genetic Resource Centre (FGSC) amongst several play individual roles in supporting the maintenance of global microbial diversity but without appropriate infrastructure on a global scale this cannot be performed effectively.

Ensuring these resources, and the vast majority yet to be isolated and identified, are available to the global scientific community for research and development presents many challenges. Not least the impacts of legislation. The Nagoya protocol, now ratified by 117 countries aims to ensure benefits that arise from use of countries genetic resource has unintendedly resulted in regulatory barriers. Conversely it provides an incentive to countries to establish capacity to effectively manage and utilise their own biological resources. Unfortunately, not all countries have the economic resources to support such activities, for these collaborative, regional or international approaches may be required. Culture collections are important facilities to help countries and scientists, source, utilise and develop microbial based projects.

The optimal preservation of fungi is of upmost importance, technologies for the preservation of most fungal organisms are well established but challenges remain for the growth and preservation of recalcitrant taxa and complex systems. Historically microbial collections, have preserved axenic cultures, however the rapid pace of scientific development necessitates collections to adapt to meet the need of the communities they serve. One particular requirement is the need to provide infrastructure to support microbiome research, which is intrinsic to many sustainable development goals traversing the fields of human health, medicine and agriculture. It must be remembered that currently it is estimated that less than 1% of the microbial communities that provide our ecosystem functionality have been isolated and grown. A paradigm shift is required in order to access, understand, enable beneficial microbial interventions and utilise the organisms in these communities; technologies are available to facilitate this and collaborative networks can enable technology and knowledge sharing.

In this paper we describe the current challenges facing culture collections, the importance of collaborative networks, building infrastructure to address these challenges and the need to underpin research and development in developing countries, whilst tackling the global challenges to meet the requirements of the research community and the impacts of legislation and the Nagoya protocol on access to biological resources.

## International networks

Over many years collaboration among international culture collections has been a foundation of progress. When collections have stable support, they are able to collaborate in scientific as well as process areas. Networks of collections have several benefits including shared best practices, advocacy, and more complete taxonomic coverage.

National and regional networks develop to promote shared interests and needs. In the USA, the US Federation for Culture Collections (USFCC) provided coordination and a venue for interaction over many years. With formal bylaws (https://web.archive.org/web/20160321225708/http://usfcc.us/bylaws.htm) and engagement with professional societies, the USFCC was successful in its mission “to further the science and practice of culture maintenance and systematic microbiology” although this was mainly among collections with health and industrial emphases. The USFCC quarterly newsletter from 1992 through 2002 are available online via the internet wayback machine (https://web.archive.org/web/20160321224428/http://usfcc.us/usfcc_newsletter.htm). Throughout its existence, the USFCC held regular business meetings, usually as a side-event at a larger society meeting and coordinated the J. Roger Porter award with the American Society for Microbiology from 1983 through 2017.

In response to this lapse in continuity and reflecting the general trend toward more applied microbiological research, researchers in genetics, biodiversity, and plant health, came together at a series of meetings sponsored jointly by the American Phytopathological Society (APS) and the US Department of Agriculture Animal and Plant Health Inspection Service (APHIS) to discuss the need for agriculturally and environmentally focused culture collection network. These meetings culminated in the publication of a white paper on the need for collections and spurred participants to submit a proposal to the US National Science Foundation for a research coordination network grant to organize “a community of ex situ microbial germplasm repositories.” Funded by the NSF Division of Biological Infrastructure in 2012, this group established the US Culture Collection Network (USCCN). Because most collections have minimal overlap, this brought together collections which historically did not interact. The diverse holdings of these collections include wild-type bacterial, fungal, algal, and chromist strains from nature, mutated strains from the classical genetics era and, increasingly, genetically modified strains from research projects. Collections of medically relevant or high-risk pathogens have not been engaged in the activities of the USCCN, although the network has been open to participation by any type of living microbe collection in the US.

In keeping with one of its explicit goals, the USCCN has worked to develop formal off-site back up and promoted the adoption of WFCC and OECD best practices. It has also allowed engagement by an active cohort of US culture collection promoters with the World Federation for Culture Collections (WFCC) and other international collection efforts such as the Global Biological Resource Centre Network. Despite the fact that the USCCN is time limited by the nature of its support, it has become the recognized public face of the US culture collection community.

By virtue of its longstanding activity and its formal establishment as a federation within the International Union of Microbiological Societies (IUMS), the WFCC has been a longstanding voice for culture collections. With over 700 registered collections and over 200 formal member collections, the WFCC is an important body to promote the interests of collections. This promotion comes through the sponsorship of the international conference on culture collections, most recently as a side-meeting at the 2017 IUMS meeting, and through its engagement with a myriad of ongoing efforts to promote appropriate utilization of microbial resources. Among these, engagement with the Secretariat of the Convention on Biological Diversity has been important in providing direction to the WFCC. In keeping with the increasing professionalism of collections, WFCC has a formal best practice guideline that is consistent with the published best practices put forward by the OECD.

The WFCC has provided a strong community in which to advance the idea of a formal global network of culture collections. The successful Global Biological Resource Centre Network demonstration project (Fritze et al. 2012) paved the way for increased collaboration among collections and demonstrated the value of international cooperation in microbial resource utilization. Meanwhile, the American Society of Investigative Pathologists developed an international network of biobanks called the International Society for Biological and Environmental Repositories (ISBER). While most participants in this network manage biobanks of tissues, cells, or environmental specimens, ISBER includes a division on environmental repositories which is anticipated to be a forum for interaction of microbial culture collections. Most US culture collections do not participate in ISBER activities as they are involved in culture collection groups such as the USCCN and do not consider themselves to be ‘biobanks’. Because many US collections (historically called a “stock centre”) hold genetic mutants either from the classical genetics or the modern molecular genetics era, environmental collections do not represent the needs or interests of these collections which are typically very strongly embedded in their respective research communities.

A network of microbial Biological Resource Centres could provide facilitated access to microbial resources under the Nagoya Protocol. To make meaningful progress in developing this network, stable long-term support is essential. Because microbial germplasm resources are inherently international and because there is no governmental support for this long-term, multinational community, a group of collection scientists have invited support by a global philanthropist to establish a network of living collections. The model for this is the over 1500 libraries established by Andrew Carnegie in the early twentieth century. The time is right for a global philanthropist to establish a formal network of living microbe collections.

The WFCC, in recognition of its over 70 years of service, and on behalf of its 200 members and 500 additional registered collections, and in recognition of the impact of microbial science, from antibiotics and anti-helminthics, to statins, anti-transplant rejection drugs, and foundational resources for biotechnology such as PCR or CRISPr, should be rightfully acknowledged!

## Impacts of the Nagoya protocol on access and benefit sharing

In Europe the European Culture Collections’ Organisation (ECCO; www.eccosite.org) has been active over the past 4 decades supporting the development of European collections and latterly supporting them in common approaches to compliance with international regulation. As a result of the voluntary requirements of the Convention of Biological Diversity to control access to genetic resources and share benefits from use the ECCO core Material Transfer Agreement for the supply of samples of biological material from public collections was drafted (https://www.eccosite.org/wp-content/uploads/2014/07/ECCO_core-MTA_V1_Febr09.pdf). Now that the Nagoya Protocol (https://www.cbd.int/abs/) is enacted in European member states through the EU Regulation (No. 511/2014), ECCO is revisiting this core text to adapt it for its member collections to share a common approach. The Microbial Resources Research Infrastructure (MIRRI) have gone a step further in producing its Access and Benefit Sharing Manual that provides detailed guidance to ensure its member microbial domain Biological Resource Centre (mBRCs) follow best practice, exercise due diligence in their activities and can follow the requirements of the Nagoya Protocol in a common way. What makes it difficult, not just for the mBRCs of Europe but for collections and microbiologists worldwide is that the Parties to the Nagoya Protocol are each putting in place regulation and requirements in accordance with their own interpretations and needs. Processes therefore differ from country to country.

The Nagoya Protocol on Access to Genetic Resources and the Fair and Equitable Sharing of Benefits Arising from their Utilization (https://www.cbd.int/abs/) is a 2010 supplementary agreement to the 1992 Convention on Biological Diversity. A visit to the ABS Clearing House (https://absch.cbd.int/) will demonstrate how countries are responding; as of July 2019, 117 countries are party to the protocol whilst only 64 of these have relevant law in place. In Europe, the EU Regulation on Access and Benefit Sharing (ABS) was enacted introducing a common approach to regulatory control and inviting due diligence in compliance practice. However, the EU regulation left it open for EU countries to independently decide if they wished to implement access control to their genetic resources. The result is that each country in Europe and beyond designs and implements its own controls. Each scientist not only needs to understand the requirements of the country in which they work but also that of the provider country of the organisms they are working with to ensure they are complying and operating within the spirit of the CBD.

Countries implementing the Nagoya Protocol require users of genetic resources to seek permission to access them in advance of collection i.e. obtain prior informed consent (PIC). To achieve this requires the negotiation of mutually agreed terms (MAT) which will include agreement on the benefits that will be shared from the intended use. Smith et al. (2017) describe how these impact on microbiology and they provide a workflow to help scientists make appropriate decisions to enable them to comply with ABS requirements. As almost half of the parties to the protocol are yet to put measures in place and many countries that are party to the CBD are yet to decide if they will adopt the protocol, it is difficult to get the information needed for compliance. Again, the ABS Clearing House provides examples of best practices some of them prescribing what to do in the interim period before law and process are introduced.

To help ensure that researchers are aware of developments and also allow them to contribute to putting in place implementable process the CBD Secretariat and the Conference of the Parties provide opportunities to input either directly or through their national authorities. Sharing of experiences and best practice is welcomed. Examples of Material Transfer Agreement, community model contractual clauses, codes of conduct, guidelines and best practices are available for use or adaption;

- European Culture Collections' Organisation (ECCO) core MTA (https://www.eccosite.org/ecco-core-mta/)
- Microbial Resources Research Infrastructure (MIRRI) ABS Manual (https://zenodo.org/record/284881)
- Global Genome Biodiversity Network (GGBCN) ABS Guidance, Best Practice for ABS: (https://www.ggbn.org/docs/ABS_Guidance/GGBN%20Guidance%20_Best_Practice_June_2015-Final.pdf)
- International Organisation for Biological Control of Noxious Animals and Plants (IOBC) Commission on Biological Control and ABS (https://www.iobc-global.org/global_comm_bc_access_benefit_sharing.html)
- Consortium of European Taxonomic Facilities (CETAF) Code of Conduct and Best practices (https://cetaf.org/sites/default/files/final_cetaf_abs_coc.pdf)

Although it is often a time-consuming process, countries such as India and Brazil have made their requirements and application processes apparent. However, many countries are still going through the process of designing their own approaches and establishing what practices best suit them. It is imperative that in doing so they look at the working examples that are in place and take note of the concerns that over restrictive regulations impede trade and science. Hopefully, the conclusions of the recent Assessment and review of the effectiveness of the Nagoya protocol (https://www.cbd.int/doc/c/7f9f/3d30/46a50d2e3f693bb57895d882/sbi-02-l-03-en.pdf) submitted to the Subsidiary Body On Implementation Second meeting in Montreal, Canada, 9–13 July 2018 will lead to improvements in the country implementation strategies.

Of relevance to biologists, is the continuing debate on whether access to Digital Sequence Information (DSI) should be treated in the same way as accessing the genetic resources (https://www.cbd.int/abs/dsi-gr.shtml). The CBD Secretariat has demonstrated the wish to secure broad input to the topic through requests for contributions to this debate through the request for: Submission of views and information and call for expression of interest to undertake studies. They also asked for nominations to the Ad Hoc Technical Expert Group on Digital Sequence Information on Genetic Resources. It is important that the right information is provided and that the most appropriate people are advising on these issues. Practical solutions are needed that facilitate science and discovery but also ensure appropriate and adequate sharing of the benefits which are normally non-monetary and rarely monetary.

## Considerations in compliance with the Nagoya protocol on access and benefit sharing

When considering the use of genetic resources (organisms) scientists must ensure they comply with provider country requirements under the Nagoya Protocol. This is in addition to checking whether other permissions are needed for example regarding CITES, quarantine, export licences etc. and land owner/ protected area access. The key elements of information required for the assessment are the location where the scientist is collecting the genetic (biological) materials from and whether access is regulated (resources should be accompanied by a Material Transfer Agreement (MTA) indicating where the material originated from and what individuals can and cannot do with it). It is important to know when the resources were, or will be collected (isolated from in situ); and to be clear of the intended use - some activities/observations do not trigger the Nagoya Protocol—e.g. identification/taxonomy but this depends on the country of origin.

A scientists first port of call is to get provider country information from the ABS Clearing House and establish if it is party to the Nagoya Protocol. If the country is not Party to the protocol, it may be Party to the CBD and have ABS measures in place. If neither is in place, no further action is required other than ensuring other permissions such as plant health permits have been obtained.

If the country is Party to the Protocol, scientists should establish what ABS measures are in place and whether these are enacted. If the genetic material (organism) was collected before October 2014 then Nagoya doesn’t apply. If the collection was after that date, and the country controls access, prior Informed Consent (PIC) must be obtained and Mutually Agreed Terms (MAT) negotiated before collection and use.

The Nagoya Protocol is triggered by utilisation—i.e. conducting R&D on the genetic and/or biochemical composition of genetic resources, including through the application of biotechnology. If the country does not control access (UK and much of the EU) scientists are free to collect and use the materials. If it is not clear whether access legislation is in place, scientists should contact the relevant national focal point and keep a record of the enquiry email and any response as this is part of the due diligence process. The fact that there may be no information on the ABSCH does not mean that the country does not have process and measures in place (https://absch.cbd.int/). Due diligence requires confirmation of country requirement, often needing consultation with country National Focal Points or Competent National Authorities (Beckett 2017). The process does not end there as often a country requires monitoring and reporting, this will differ from country to country. An example is seen in the information provided by the National Biodiversity Authority in India (https://nbaindia.org/), specifically see the Operational Guidelines to the State Biodiversity Boards for Processing of Applications for Access to Biological Resources received under Sect. 7 of the Biological Diversity Act, 2002 (https://nbaindia.org/uploaded/pdf/Guidelines_for_Processing_ABSapplications_SBBs.pdf). These require periodical reports from the applicant about the activities carried out on the accessed biological resources and evidence that the agreed benefits have been shared. Due diligence declarations are required in the UK, information and guidance is given (https://www.gov.uk/guidance/abs). Provider countries will need to be contacted to discover their requirements if they are not provided on the ABSCH via the country profiles.

In summary, while the spirit of the Nagoya protocol helps developing countries protect their biodiversity by ensuring access and benefit sharing, there is no provision to help them preserve and store their own biodiversity in country. This is a deficit that requires attention, as without it their biodiversity will either not be preserved or they will have to continue to rely on overseas collections in developed countries. For some countries, such as India and Brazil who have established strict control over access to, and export of, their natural resources, the very regulations designed to protect their resources could actually inhibit research and development for the benefit of their indigenous populations.

## Availability of technologies for preservation

Existing preservation techniques for fungi have been designed for well-equipped laboratories in major national service culture collections. There is a plethora of methods described in the literature for the storage and ex situ conservation of fungi in axenic culture which range from basic methods suitable for application in the short term (up to 2 years) through to long term methods that allow for many years (50+ ) of storage. The pros and cons of using preservation methods were previously evaluated by Ryan et al. (2000) who devised a decision-based key, which incorporated logistical, scientific and financial criteria to aid scientists in the selection of preservation regime.

Basic methods which are often economically cost effective requiring only basic infrastructure, are well described and include techniques such as serial sub-culture (Smith and Onions 1994), and storage in oil (Fennell 1960; Smith et al. 2001), water (Burdsall and Dorworth 1994; Boeswinkel 1976) or in sterile soil (Smith et al. 2001; Booth 1971). However, the principle aim of preservation is to maintain purity, viability, genomic integrity and avoid the selection of variants from within a population and lessen the prospects of strain degradation (Ryan and Smith 2004). These ‘basic’ methods will not provide this due to selection pressures incurred during storage and maintenance and the risks of loosing collections due to intermittent electricity supply, contamination and the availability of staff resources.

For most scientists, the preservation methods of choice for long-term, stable storage of important cultures remain to be freeze-drying (Ryan and Smith 2004; Tan 1997) and cryopreservation at ultra-low temperature (Ryan and Smith 2004; Verkleij et al. 2015). These methods have been used for over fifty years and are now commonly applied where infrastructure and resources allow. However, it is not necessarily practical or indeed possible to use the more technical, resource intensive methods to be used in collections in LMIC countries.

Freeze drying which requires the purchase of expensive equipment, is used routinely for sporing fungi. Once preserved, cultures are easy to handle and occupy little storage space with a proven shelf life of up to 50 years or longer (Smith and Onions 1994). Ampoules can also be circulated to scientists, throughout the world, without having to be revitalised beforehand, cutting the cost of courier charges and avoiding potential damage to the organism during transport if it was transported active on agar. This is advantageous to scientists in LMIC countries as they can get their cultures freeze dried overseas and have a working stock of ampoules returned to them for routine use. Although ideal, a refrigerated or consistent storage temperature is not critical for vacuum sealed ampoules, and working stocks can be produced for routine activities, negating the need for frequent serial transfer. However, as no one preservation method can be 100% guaranteed to be successful for all culturable fungi (Ryan and Smith 2004), good practice dictates the use of a least two preservation methods, typically cryopreservation and lyophilisation. While this may increase costs, it does provide opportunity for scientists to get their resources backed up as a disaster mitigation strategy.

Cryopreservation has become the pre-eminent methodological approach for the preservation of fungi in major international public service culture collections (Ryan and Smith 2004). Reports of cryopreservation of specific species or groups of fungi are often published. Recent examples include methods for ectomycorrhiza (Crahay et al. 2012), arbuscular mycorrhizae (Lalaymia et al. 2014) and Hop Powdery Mildew (Wada and Reed 2017). It is the ‘gold standard’ as the chances of strain drift are significantly less than other approaches if samples are correctly cooled and stored. However, cryopreservation infrastructure is very expensive, cryo storage vessels, controlled rate coolers, safety equipment and nitrogen supply can be costly (Ryan et al. 2000). There are basic apparatus available that can achieve the recommended controlled cooling rate of − 1 °C min^−1^ with a suitable cryoprotectant such as the commercial ‘Mr Frosty’ (Nalgene, USA) OR Cryocell cooler, although reproducibility and effectiveness of preservation is best served using chamber-controlled rate cooling. Cryopreservation in mechanical freezers is not recommended as samples are at risk in the event that electricity supply is compromised and temperatures warmer than − 133 °C can result in the recrystallisation of ice which can damage cells.

Modern advances in cryotechnology may have application. Controlled rate cooling can be achieved without the need for a refrigerant gas such as the Stirling cycle cooling approach (Ryan et al. 2014); while N2 free cold-chain technology is being developed. Similarly, preservation technology, has seen the introduction of vitrification (Tan and Stalpers 1996) and AE (alginate encapsulation) cryopreservation (Ryan 2001; Benson et al. 2018) which negate the need for the use of controlled rate cooler and bring down costs. However, even in high income countries, few collections are actively involved in preservation protocol research and development and where it is employed it is often the result of technology transfer from biomedical field.

## New and future challenges for microbial preservation

Challenges remain for the vast number of unculturable organisms, where suitable procedures for their isolation and culture do not exist. Culture collections (with only minor exceptions for example the provision of mycoparasites) do not supply mixed cultures, microbial consortia or microbiome samples. The is primarily due to the lack of an effective cryopreservation regime to optimally store such samples, with no collection having the capacity to routinely store and supply microbiomes. One goal is to maintain the functionality of cryopreserved microbiome, Bell (2019) used the analogy ‘If you try and take a cat apart to see how it works, the first thing you have on your hands is a nonworking cat’ (Adams 2002) and this is an important consideration when trying to conserve a microbiome sample. The main reason for this is that when samples are cryopreserved, only the freeze tolerant components of the microbial community will survive, causing a disturbance of the microbial equilibrium of the wild-type sample.

There are few reports of successful microbial consortia cryopreservation. Kerckhof et al. (2014) evaluated a cryopreservation protocol that succeeded in preserving both community structure and functionality of microbiomes, while Vekeman and Heylen (2015) described methods for the cryopreservation of mixed communities but only at − 80 °C. Because of an international focus on the critical importance of the plant and human microbiome, and the significance of the fungi within these systems, biological resource centres need to focus efforts to develop new and improved methodologies for the storage of microbiome samples. For fungi, this specifically includes endophytes, mycorrhizae, symbiotic mutualists and associated organisms many of which will have commercial applications as bioinoculants. It is important for scientists researching these novel preservation methods to disseminate their findings and equip peers in LMIC countries through research collaborations in order to establish infrastructure and ensure long term sustainability.

## Microbial collections supporting sustainable development goals

Fungal Culture collections through the application of science, technology and knowledge generation related to their resources are well placed to support the bioeconomy and to meet society’s Grand Challenges in addressing several of the UN’s key Sustainable Development Goals (SDG) including zero poverty (SDG1), zero hunger (SDG2), good health and well-being (SDG3), quality education (SDG4), industry innovation and infrastructure (SDG9), life below water (SDG14) and life on land (SDG15) (Griggs et al. 2013; Waage et al. 2015).

There is wide recognition that harnessing the full value of fungal biological resources and processes provides incentive to generate solutions from the development of new products and services (Table 3) within a more resilient, eco-efficient bioeconomy in order to meet the SDG’s.

**Table 3:** Sustainable Development Goals, Targets and Solutions

Goal	Target	Examples of potential solutions (fungi and fungal culture collections)
SDG 2: zero hunger	Use fungi to contribute indirectly towards ending hunger	Microbiome based solutions to increase crop yield Development of mycoproteins Biocontrol Solutions Fungi as reference strains
SDG 3: good health and well-being	Use fungi and knowledge generation to provide solutions for ensuring good health	Development of nutraceuticals Fungi as a source of novel compounds for drug development e.g. antimicrobials Fungi as a source of alternatives for chemicals e.g. biofertiliser
SDG 6: clean water and sanitation	Use and knowledge of novel fungi in water systems to aid sustainable management of water systems for improved sanitation	Knowledge of fungi in water systems Fungi used in bioremediation of waste and pollutants Fungi as reference strains Fungi in Water systems
SDG 7 affordable and clean energy	Use of fungi to produce novel fuels and knowledge to prevent losses in fuel systems	Novel biofuels for example the application of lignocellulosic degrading fungi Fungi as biodeteriogens of fuel
SDG 15: life on land	Use of fungi, particularly relationship with Plant life	Resource Centres for conversation of fungi as: indicators of climate change habitat regeneration Biofertilizers Sustainable production of crops Control of Invasive species

The importance of institution and infrastructure goals in meeting the SDG’s has been reported (Waage 2015) and fungal culture collections can be part of a broader strategy to achieve them. It is necessary to create infrastructure globally to organise fungal resource culture collections, share technology and know-how and to target specific activities in collaboration. Individual collections, even those in higher income countries, do not have the capacity to provide solutions for global microbiological needs. This requires a coordinated approach by a consortium of national collections (for example through the WFCC or the European Research Infrastructures such as MIRRI) guided by their stakeholders and in association with stakeholders in LMIC’s. No single country currently offers a complete coverage of microbial diversity and associated services.

## Supporting infrastructure in fungal taxonomy—MycoBank

The scientific name of an organism is the primary key to access information on structure, properties and other aspects of its biology. Since January 2013, registration of new fungal names is a mandatory requirement for valid publication under the International Code of Nomenclature for algae, fungi and plants (ICN) (McNeill et al. 2012). Three online repositories were designated to serve for the formal registration of new fungal names: Fungal Names, Index Fungorum and MycoBank. Besides the new name of a fungus a set of core data needs to be provided including information on place of publication and deposit of the type specimen (as dried specimen in a fungarium or as a living culture preserved in a metabolically inactive state in a culture collection). The unique database registration number allocated for the name is to be cited in the place of publication for the name to be valid.

MycoBank was launched in 2004 as an online open access repository with the primary aim to register all fungal taxonomic novelties including new names and combinations (“MB” number), and provide descriptions and illustrations (Crous et al. 2004; Robert et al. 2013). While initially set up by mycologists of the Westerdijk Fungal Biodiversity Institute (then still named CBS-KNAW), MycoBank was brought under the auspices of the International Mycological Association (IMA) in 2010, underpinning it’s the global significance. In 2018 MycoBank attracted 440,000 unique end users. The database runs on the BioloMICS software (Robert et al. 2011) and is regularly updated and improved to serve the needs of end users and curators of MycoBank. In 2013 an important function was added which allows for the registration of later typification events (“MBT” number) such as the designation of lecto-, epi- or neotypes. These events are normally difficult to trace in the literature but should be easy to find to avoid that authors would independently propose typifications for the same names based on different specimens, and thus only would create more confusion instead of definitively settling the applications of the name.

Mycobank is a community-driven online repository striving to reach completeness, avoid redundance and, while offering free and widely used services it is being upheld without any financial support other than from its hosting organisation (Westerdijk Institute). A major challenge for any online repository of biodiversity data is to correctly link species (and subsequently higher taxonomic ranks) with sequence data and this can only be done via specimens (dried specimens, living strains) (Durães Sette et al. 2013; Robert et al. 2013). BRCs and other collections such as fungaria preserve these biological reference materials and collect and store associated information (Verkleij et al. 2015).

Ever since DNA sequencing was introduced as a routine tool in fungal taxonomy it has greatly changed classification, improved identification, and deepened our understanding of the diversity of the fungal microbiomes as cryptic taxa were revealed. New subclades sometimes proved to differ markedly in physiology or pathogenicity (Crous et al. 2015; Minnis 2015), and helped in more accurately recognizing relevant entities (often recognized as new species) like human or plant pathogens that need to be controlled or regulated and not mistaken for their harmless relatives (Bonants et al. 2013; Irinyi et al. 2015). Many genera of fungi proved poly- and or paraphyletic. Although many new generic names have been proposed for the clades in recent years, the proportions of genus names actually linked to validated DNA data is still relatively low, but efforts are being made to improve the situation. Well-characterized cultures preserved in mBRCs are being selected to serve as types for such genera. Correct identification is critical as misidentifications lead to unnecessary and costly efforts in research and management, economic losses and even societal risks (Hawksworth 2015). Since the first DNA sequences for fungi became available in GenBank in the early 1990s, large numbers of sequences have been deposited including sequences based on incorrectly identified material or for which no voucher material is available, confronting users blasting their unknown sequences against these resources with huge problems.

It is good practice to deposit type- or other correctly identified voucher material in public collections where it is preserved according to highest standards and can be obtained by other scientists as reference for further work (Schoch et al. 2014; Vu et al. 2016). DNA barcoding approaches are based on such traceable sources, where relatively short and readily amplifiable loci (the “barcodes”) serve as marker sequences. DNA barcodes have proven to be very useful for diagnostics in human and plant health, food security and applied sciences (Hebert et al. 2003; Yahr et al. 2017). For fungal identification, the Internal Transcribed Spacer (ITS) region of nuclear ribosomal DNA has been most used, and it is still the only official DNA Barcode locus for fungi (Schoch et al. 2012), although other loci have gained significance for identification in certain fungal groups where ITS provides insufficient resolution (Stielow et al. 2015). BRCs have played an important role in generating fungal barcodes. Vu et al. (2016, 2018) presented an analysis of a major barcoding effort for yeasts and filamentous fungi preserved in the CBS Collection, marking the release of 8700 and 24,000 sequences of type-and other reference strains of yeast and filamentous fungal species, respectively. These releases constitute a huge step forward in coverage of fungal biodiversity through reference sequences. Such data not only are of great value for strain identification, but also facilitate the selection of strains for whole genome sequencing projects, for which BRC holdings are an increasingly important source of material.

On a global scale the capacity of the collections to accept new deposits (such as type strains for new fungi) is insufficient and major investments are necessary, especially in LMIC with often rich biodiversity but lacking funds to establish collections or develop existing ones. Creating capacity in these regions is considered of great urgency as it would be most practical under ABS legislation to implement the Nagoya Protocol.

The linking of organism names in all curated nomenclatural databases to other sources of scientific information is required to more effectively find information for scientific analyses and management in health care, food security and conservation. For MycoBank structural links have been created to several websites including Catalogue of Life (CoL), Encyclopaedia of Life (EOL), Global Biodiversity Information Facility (GBIF), Integrated Taxonomic Information System (ITIS), Google Scholar, PubMed, Wikispecies, Barcoding of Life Database (BOLD) Systems, EMBL, NCBI, All Russian Collection of Microorganisms (VKM), CBS collection, Global Catalogue of Microorganisms (GCM).

## Supporting infrastructure: bioinformatics

Sciences are evolving at an incredible pace and many paradigm changes have been observed in the last decades in all scientific fields. The same is of course true in biology where the introduction of high throughput technologies allows to generate incredible amounts of interesting data that nobody would have been able to imagine even 10 to 20 years ago. DNA sequencing, for example, has certainly been one of the most striking revolutions over last few decades since the discovery of its existence. 20–30 years ago, it was a challenge to sequence a few hundred base pairs and it’s now relatively easy and cheap to sequence complete genomes. It was impossible to predict that one could sequence an entire human genome in a few hours for less than 1000 US$. The consequences of all these new developments are huge in terms of:

1. Amounts of data to be stored, handled and analysed
2. Diversity of data types
3. Algorithms that need to be developed and tuned to be able to properly analyse complex and diverse datasets
4. Interconnection of datasets
5. Formats, reusability of data and data exchange
6. Target audience of the produced data, for humans or for machines?
7. The rise of artificial intelligence and its consequences
8. The costs associated with the points mentioned above and the shortage of software developers, database specialists or data analysts (to mention only a few) and their associated high salaries
9. Building large multi-disciplinary teams (biologists, ecologists, bioinformaticians, software, algorithms or databasing specialists, etc.)

Data storage, analysis, distribution and use have been addressed over the years in several European funded projects and global initiatives. Most recently, the Microbial Resources Research Infrastructure (MIRRI) has published its activities in this area; it is anticipated that on the establishment of its legal entity as a European Research Infrastructure Consortium it will initiate the construction of its information system (Casaregola et al. 2016). There has been significant input from the World Data Centre for Microorganisms (https://www.wdcm.org/) who have been improving how mBRC data is presented and linked to other data sources to enhance and enrich information available on microbial strains (Wu et al. 2017). There are many separate data sources of relevance to the use and characterisation of microorganisms which require the collaboration, coordination, mechanisms for interoperability and their dynamic linking to benefit the user and enable innovation. It requires the mBRC community to join forces and work with research infrastructures such as ELIXIR (https://elixir-europe.org/) who unite Europe’s leading life science organisations in managing and safeguarding the increasing volume of data being generated by publicly funded research. ELIXR coordinates, integrates and sustains bioinformatics resources across its member states and enables users in academia and industry to access services that are vital for their research. Another example of a valuable partner in such a relationship is EU-OPENSCREEN (https://www.eu-openscreen.eu/) who integrate high-capacity screening platforms throughout Europe, which jointly use a rationally selected compound collection, comprising up to 140,000 commercial and proprietary compounds collected from European chemists. The European Marine Biological Research Infrastructure Cluster (EMBRIC—https://www.embric.eu/) have demonstrated how such research infrastructures can be brought together with researchers and resource holders such as mBRCs to offer targeted research and solutions to bioindustry (Brennecke et al. 2018; Pina et al. 2018). Very few LMIC’s have either the infrastructure or resource to invest in the physical servers for storage and computation, and the bioinformaticians needed to undertake the data-analysis. Without these, they will continue to rely on existing global capacity such as services provided through the European Bioinformatics Institute (EMBL), GenBank (NIH genetic sequence database) and the training opportunities that these European and North American institutions offer.

## Discussion—summary and recommendations

Harnessing fungi for use by humankind to enhance bioeconomies in LMIC’s is pre-requisite. While existing global fungal culture collections such as CABI and CBS provide a mechanism for LMIC’s to deposit their strains, there is a pressing requirement for capacity to be established ‘in country’ to empower counties to be able to look after their most important and valuable national biodiversity and microbiological assets. A more urgent challenge remains in finding funding for collections to underpin research and good science which compete against perceived greater priorities such as food security or academic research. Innovative funding mechanisms and efficiencies in coordination and management are required to secure the microbial resources on which these depend (Smith et al. 2014). Therefore, it is essential that future initiatives build on the best of experiences already established.

The immediate challenges for the global BRC community are to empower and equip LMIC’s so that they are able to maintain their biodiversity and handle their data, this can be achieved through:

- Establishment of capacity at national or regional level, respecting local laws and international legislation
- Enhancement of international collaboration within legislative parameters
- Investment in new technology approaches ( = technology transfer)
- Provision of training in taxonomy, data handing, bioinformatics, biological resource technology and management
- Establishing national priority lists to ensure key national assets are preserved for example disease reference, industrial and agricultural strains
- Provision of support to develop commercially important strains including antimicrobials, bio inoculants for Agritech, biocontrol agents and food strains
- Engagement with stakeholders involved in microbiome research to underpin this critical area of research and the commercial opportunities arising from it
- Investigate innovative funding sources

International networks such as the WFCC are well placed to provide an overview of global capacity, but a concerted effort between donors, government and industrial/commercial stakeholders is required to establish capacity in order to meet SDG’s and national priorities. Furthermore, the Nagoya Protocol should not be a barrier to international sharing of strains for research and development. Indeed, if countries have their own mechanisms to store and control their national resources, then ensuring the equitable sharing of benefits arising from their exploitation should be achievable though greater transparency and traceability and reduce the concerns of biopiracy. For countries where that is not possible, International culture collections which operate best practice with respect to the Nagoya Protocol should prevent unregulated bioprospecting and reassure depositors from LMIC countries.

## References

[CR1] Adams D (2002). The Salmon of doubt: hitchhiking the galaxy one last time.

[CR2] Beckett K (2017). Understanding access and benefit sharing. Biologist.

[CR3] Bell T (2019). Next-generation experiments linking community structure and ecosystem functioning. Environ Microbiol Rep.

[CR4] Benson EE, Harding K, Ryan M, Petrenko A, Petrenko Y, Fuller B (2018). Alginate encapsulation to enhance biopreservation scope and success: a multidisciplinary review of current ideas and applications in cryopreservation and non-freezing storage. Cryo Lett.

[CR5] Boeswinkel HJ (1976). Storage of fungal cultures in water. Trans Brit Mycol Soc.

[CR6] Bonants P, Edema M, Robert V (2013). Q-bank, a database with information for identification of plant quarantine plant pest and diseases. EPPO Bull.

[CR7] Booth C (1971). Fungal culture media. Method Microbiol.

[CR8] Brennecke P, Ferrante MI, Johnston IA, Smith D (2018). A collaborative European approach to accelerating translational marine science. ICES J Mar Sci Special Issue Methodologies for Outreach in the Marine Sciences. J Mar Sci Eng.

[CR9] Burdsall HH, Dorworth EB (1994). Preserving cultures of wood decaying Basdiomycotina using sterile distilled water in cryovials. Mycologia.

[CR10] Casaregola S, Vasilenko A, Romano P, Robert V, Ozerskaya S, Kopf A, Glöckner FO, Smith D (2016). An Information System for European culture collections: the way forward. SpringerPlus.

[CR11] Crahay C, Declerck S, Colpaert JV, Pigeon M, Munaut F (2012). Viability of ectomycorrhizal fungi following cryopreservation. Fungal Biol.

[CR12] Crous PW, Gams W, Stalpers JA, Robert V, Stegehuis G (2004). MycoBank: an online initiative to launch mycology into the 21st century. Stud Mycol.

[CR13] Crous PW, Hawksworth DL, Wingfield MJ (2015). Identifying and naming plant-pathogenic fungi: past, present, and future. Ann Rev Phytopathol.

[CR14] Durães Sette L, Pagnocca FC, Rodrigues A (2013). Microbial culture collections as pillars for promoting fungal diversity, conservation and exploitation. Fungal Genet Biol.

[CR15] EU Regulation on ABS (EU) No 511/2014 https://eur-lex.europa.eu/legal-content/EN/TXT/?uri=CELEX:32014R0511

[CR16] Fennell DI (1960). Conservation of fungus cultures. Bot Rev.

[CR17] Fritze D, Martin D, Smith D (2012) Final report on the GBRCN Demonstration Project. Germany: GBRCN Secretariat. ISBN 978-3-00-038121-8.

[CR18] Griggs D, Stafford-Smith M, Gaffney O, Rockström J, Öhman MC, Shyamsundar P, Steffen W, Glaser G, Kanie N, Noble I (2013). Sustainable development goals for people and planet. Nature.

[CR19] Hawksworth DE (2015). Naming fungi involved in spoilage of food, drink, and water. Curr Opin Food Sci.

[CR20] Hebert PDN, Cywinska A, Ball SL, DeWaard JR (2003). Biological identifications through DNA barcodes. Proc R Soc Lond Biol.

[CR21] Irinyi L (2015). International Society of Human and Animal Mycology (ISHAM)-ITS reference DNA barcoding database—the quality controlled standard tool for routine identification of human and animal pathogenic fungi. Med Mycol.

[CR22] Kerckhof FM, Courtens EN, Geirnaert A, Hoefman S, Ho A, Vilchez-Vargas R, Pieper DH, Jauregui R, Vlaeminck SE, Van de Wiele T, Vandamme P, Heylen K, Boon N (2014). Optimized cryopreservation of mixed microbial communities for conserved functionality and diversity. PLoS ONE.

[CR23] Lalaymia I, Cranenbrouck S, Declerck S (2014). Maintenance and preservation of ectomycorrhizal and arbuscular mycorrhizal fungi. Mycorrhiza.

[CR24] McNeill J, Barrie FR, Buck WR, Demoulin V, Greuter W, Hawksworth DL, Herendeen PS, Knapp S, Marhold K, Prado J, Prud’homme van Reine WF, Smith GE, Wiersema JH, Turland NJ (2012). International Code of Nomenclature for algae, fungi, and plants (Melbourne Code) adopted by the Eighteenth International Botanical Congress Melbourne, Australia, July 2011. [Regnum Vegetabile No. 154.].

[CR25] Meryman HT, Williams RJ, Douglas M, St J (1977). Freezing injury from "solution effects" and its prevention by natural or artificial cryoprotection. Cryobiology.

[CR26] Minnis Andrew M. (2015). 7 The Shifting Sands of Fungal Naming Under the ICN and the One Name Era for Fungi. Systematics and Evolution.

[CR27] Piña Mery, Colas Pierre, Cancio Ibon, Audic Annie, Bosser Lucas, Canario Adelino, Gribbon Philip, Johnston Ian A., Kervella Anne Emmanuelle, Kooistra Wiebe H. C. F., Merciecca Matthieu, Magoulas Antonios, Nardello Ilaria, Smith David, Pade Nicolas, Robinson Douglas, Schoen Antoine, Schultz Fanny, Kloareg Bernard (2018). The European Marine Biological Research Infrastructure Cluster: An Alliance of European Research Infrastructures to Promote the Blue Bioeconomy. Grand Challenges in Marine Biotechnology.

[CR53] Robert V, Szoke S, Jabas B, Vu D, Chouchen O, Blom E, Cardinali G (2011). BioloMICS software: biological data management, identification, classification and statistics. Open Appl Inform J.

[CR28] Robert V (2013). MycoBank gearing up for new horizons. IMA Fungus.

[CR29] Ryan MJ (2001). The use of immobilisation for the preservation of *Serpula lacrymans*. Mycologist.

[CR31] Ryan MJ, Smith D (2004). Fungal genetic resource centres and the genomic challenge. Mycol Res.

[CR32] Ryan MJ, Smith D, Jeffrie P (2000). A decision-based key to determine the most appropriate protocol for the preservation of fungi. World J Microbiol Biotechnol.

[CR30] Ryan MJ, Kasulyte-Creasey D, Kermode A, Phue San S, Buddie AG (2014). Controlled rate cooling of fungi using a Stirling cycle freezer. Cryo-Lett.

[CR34] Schoch CL, Seifert KA, Huhndorf S, Robert V, Spouge JL (2012). Nuclear ribosomal internal transcribed spacer (ITS) region as a universal DNA barcode marker for Fungi. Proc Natl Acad Sci USA.

[CR33] Schoch CL, Robbertse B, Robert V, Vu D, Cardinali G et al (2014) Finding needles in haystacks: linking scientific names, reference specimens and molecular data for Fungi. Database 2014 10.1093/database/bau061. PMC4075928 24980130

[CR35] Sharma A, Shouche Y (2014). Microbial Culture Collection (MCC) and International Depositary Authority (IDA) at National Centre for Cell Science, Pune. Indian J Microbiol.

[CR38] Smith D, Onions AHS (1994) The Preservation and Maintenance of Living Fungi, second edition IMI Technical Handbooks 2: Wallingford, CAB International.

[CR40] Smith D, Thomas VE (1998). Cryogenic light microscopy and the development of cooling protocols for the cryopreservation of filamentous fungi. World J Microbiol Biotechnol.

[CR36] Smith D, Ryan MJ, Day JG (eds) (2001) The UK National Culture Collection Biological Resource: Properties, maintenance and management. pp 382. UK National Culture Collection, Egham.

[CR52] Smith D, McCluskey K, Stackebrandt E (2014). Investment into the future of microbial resources: culture collection funding models and BRC business plans for biological resource centres. SpringerPlus.

[CR37] Smith D, Lyal C, da Silva M, Jackson J (2017). Explanation of the Nagoya Protocol on Access and Benefit Sharing and its implication for microbiology. Microbiology.

[CR39] Smith D, Hinz H, Mulema J, Weyl P, Ryan MJ (2018). Biological control and the Nagoya Protocol on access and benefit sharing – a case of effective due diligence. Biocontrol Sci Techn.

[CR41] Stielow JB (2015). One fungus, which genes? Development and assessment of universal primers for potential secondary fungal DNA barcodes. Persoonia.

[CR42] Tan CS, Stalpers JA (1996) Vitrification of fungi. In: Proceedings, International Biodiversity Seminar ECCO XIV Meeting June 30–July 4, 1995, Gozd Martuljek, Slovenia. Ljubljana: Slovenian National Committee for UNESCO and the National Institute of Chemistry.

[CR43] Vekeman Bram, Heylen Kim (2015). Preservation of Microbial Pure Cultures and Mixed Communities. Springer Protocols Handbooks.

[CR44] Verkleij GJM, Rossman A, Crouch JA (2015) The role of herbaria and culture collections. In: McLaughlin DL, Spatafora JW (eds) The Mycota Vol VII (B): Systematics and Evolution Springer, New York, p 205–225

[CR45] Vu D, Groenewald M, Szöke S, Cardinali G, Eberhardt U, Stielow B, de Vries M, Verkleij GJM, Crous PW, Boekhout T, Robert V, Samson RA (2016). DNA barcoding analysis of more than 9 000 yeast isolates contributes to quantitative thresholds for yeast species and genera delimitation. Stud Mycol.

[CR46] Vu D, Groenewald M, de Vries M, Gehrmann T, Stielow B, Eberhardt U, Al-Hatmi A, Groenewald JZ, Cardinali G, Houbraken J, Boekhout T, Crous PW, Robert V, Verkleij GJM (2018). Large-scale generation and analyses of filamentous fungal DNA barcodes boosts coverage of the kingdom fungi and reveals thresholds for fungal species and higher taxon delimitation. Stud Mycol.

[CR47] Waage J, Banerji R, Campbell O (2015). The millennium development goals: a cross-sectoral analysis and principles for goal setting after. Lancet.

[CR48] Wada S, Reed BM (2017). Hop powdery mildew (*Podosphaera macularis*) spore cryopreservation. Cryo Lett.

[CR49] Wingfield MJ, De Beer ZW, Slippers B, Wingfield BD, Groenewald JZ (2012). One fungus, one name promotes progressive plant pathology. Mol Plant Pathol.

[CR50] Wu L, Sun Q, Desmeth P, Sugawara H, Xu Z, McCluskey K, Smith D, Alexander V, Lima N, Ohkuma M, Robert V, Zhou Y, Li J, Fan G, Ingsriswang S, Ozerskaya S, Ma J (2017). World data centre for microorganisms: an information infrastructure to explore and utilize preserved microbial strains worldwide. Nucleic Acids Res.

[CR51] Yahr R, Schoch CL, Dentinger BTM (2016). Scaling up discovery of hidden diversity in fungi: impacts of barcoding approaches. Phil Trans R Soc B.

